# Brain-Derived Neurotrophic Factor as a Clinical Biomarker in Predicting the Development of Post-Stroke Depression: A Review of Evidence

**DOI:** 10.7759/cureus.15662

**Published:** 2021-06-15

**Authors:** Dan Shan, YuanDian Zheng, Karen Froud

**Affiliations:** 1 Department of Biobehavioral Science, Teachers College, Columbia University, New York, USA; 2 College of Osteopathic Medicine, Kansas City University, Kansas City, USA

**Keywords:** brain-derived neurotrophic factor, post-stroke depression, stroke, depression, biomarker

## Abstract

Studying the relationship between brain-derived neurotrophic factor (BDNF) and post-stroke depression (PSD) may help determine the potential for depression in stroke patients at the earliest stage possible. Current research has identified changes in BDNF levels in PSD patients. Thus, this article was intended as a review of evidence with respect to changes in the expression of BDNF in patients with PSD by integrating extant findings.

We conducted a search in the electronic databases PubMed, EMBASE, and PsycINFO (all records from January 1, 2000, through October 20, 2020) using keywords: “brain-derived neurotrophic factor OR BDNF,” “post-stroke depression OR PSD,” “expression level,” “association,” and “relationship.” Returned articles were considered for inclusion in this review if they were empirical studies investigating the association between BDNF expression and PSD.

Seven original papers were selected for review and revealed inconsistent findings. Five out of seven studies reported a significant decrease in BDNF levels in PSD patients at a certain stage (most likely the early stage) of stroke after admission, whereas the other two showed contrasting findings.

Overall, this review reveals associations between changes in serum BDNF levels and depression following stroke. Whether serum BDNF levels, especially in the early phase of stroke, can be a potentially effective biomarker for predicting the risk of subsequent PSD development is still open to debate.

## Introduction and background

Post-stroke depression (PSD) is a common neuropsychiatric complication associated with stroke, whereby patients gradually develop depressive states as they are recovering from stroke sequelae [1]. Given that the pathophysiology of PSD remains unclear and the outcomes of PSD can dramatically and widely affect the life quality of patients [2], it is important to find effective biomarkers for predicting the development of depression after stroke.

Brain-derived neurotrophic factor (BDNF), one subtype of neurotrophin (nerve growth factors), has drawn extensive attention from stroke researchers. Indeed, lacking the beneficial effects of BDNF in promoting neurogenesis may be related to PSD [3]. BDNF interacts with several neurotransmitters, including serotonergic, glutamatergic, and GABAergic neurotransmission that are implicated in the underlying mechanisms of depressive disorders [4-7]. In addition, animal studies [8-9] have revealed a strong association between decreased BDNF levels and increased post-stroke depressive-like behaviors, and lowered serum BDNF levels were reported in stroke human patients [10], supporting the hypothesis that BDNF may be related to PSD. Accordingly, it seems that the dysexpression of BDNF is involved in the emergence and development of PSD.

However, to date, there is scant review literature available that investigates the association between PSD and BDNF. Thus, the aim of this article was to review evidence of the relationship between BDNF and PSD in human patients, to determine whether BDNF levels might have utility as a novel potentially clinically applicable biomarker for predicting the subsequent development of PSD at a certain stage after stroke.

## Review

Method

Search Strategy

A comprehensive literature search was performed to identify relevant papers that studied the association between the expression level of BDNF and the development of PSD. Three databases, PubMed, EMBASE, and PsycINFO, were used to search studies published in English from January 1, 2000, to 20 October 2020. Search keywords were “brain-derived neurotrophic factor OR BDNF,” “post-stroke depression OR PSD,” “expression level,” “association,” and “relationship.” Dependent on the protocols in each database, keywords were adjusted and entered all together or in combinations of at least two at a time. The reference lists of each paper retrieved were subjected to manual search to identify supplementary papers not identified by the databases above. In addition, where systematic reviews using meta-analysis or other statistical methods to integrate the data of several empirical research studies were identified, the original empirical articles were retained in case of circular demonstration of research results.

Inclusion Criteria

Studies were included for review if they met all the following criteria: (1) published research articles investigating the association between BDNF and PSD or post-stroke depressive mood in recent two decades; (2) study participants were adults (≥18 years of age), with clinical diagnoses of PSD or evaluations of depression scores conducted in stroke patients, and objective assessments of BDNF levels performed for all participants; (3) changes in BDNF levels in stroke patients were clearly described; (4) peer-reviewed articles published in English. There were no restrictions on the basis of gender, sample size, duration of follow-up, or the nationality of participants.

Exclusion Criteria

Studies were excluded if they had any of the following restrictions: (1) identical articles (duplicates) from different databases; (2) systematic or narrative review articles; (3) participating patients with a history of any severe psychiatric disorder; (4) studies of mixed populations (such as stroke populations combined with other central nervous system disorders, or traumatic brain injury, or severe physical disabilities).

Results

Numbers of Studies Included/Excluded

**Figure 1:**
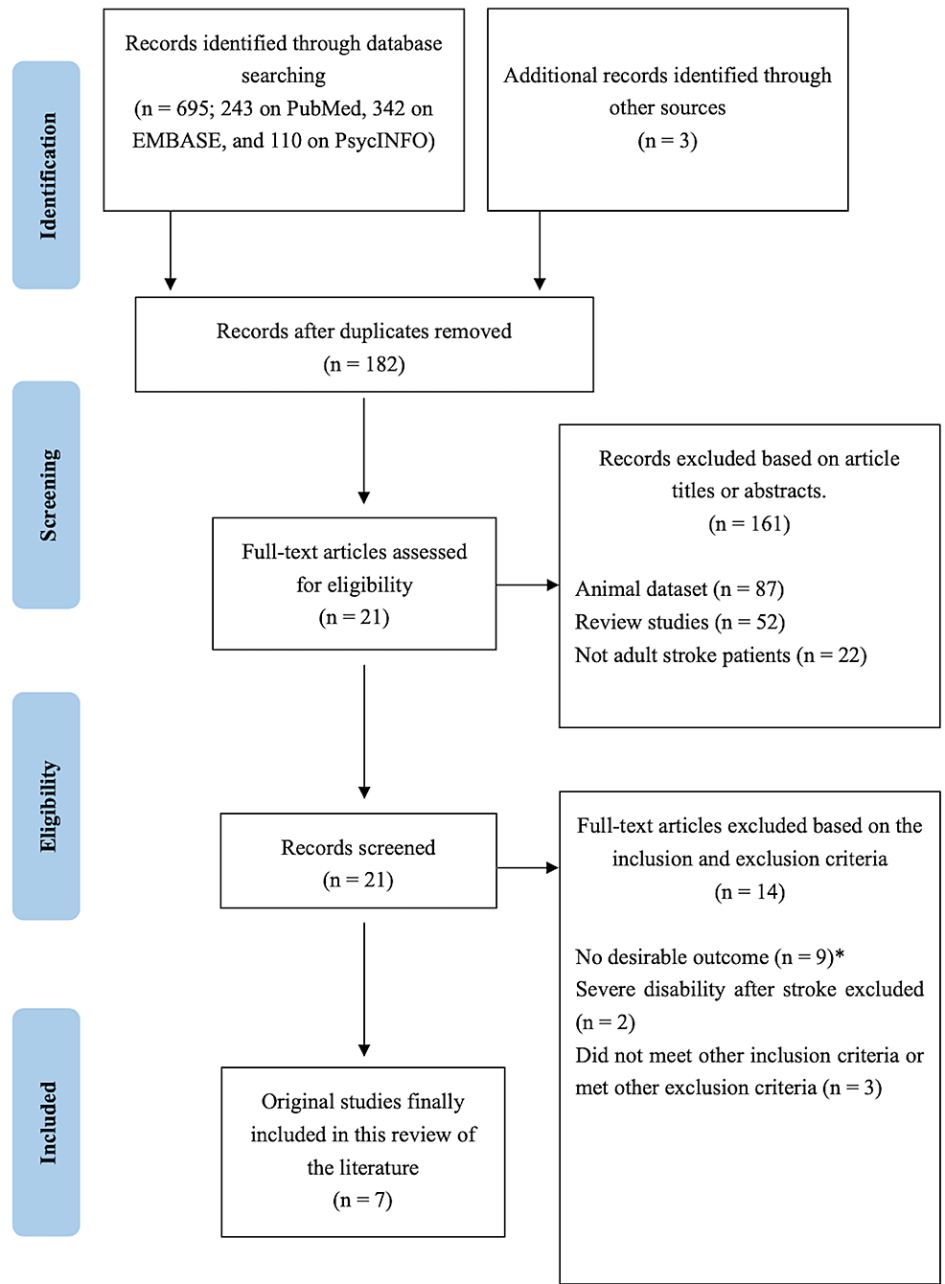
Flow Diagram of the Literature Review Process Note. *Desirable outcome: both BDNF and PSD mentioned in full texts of extracted articles, and the association clearly indicated as a conclusion of the study. Abbreviations: BDNF, brain-derived neurotrophic factor; PSD, post-stroke depression

A total of 698 references were found through the electronic literature search. Of these, 516 were identified as duplicates and excluded. Thus, 182 were initially evaluated. Another 161 were excluded based on information in the titles or abstracts. In the full-text review stage, 21 papers were carefully screened, and finally, seven studies were chosen for discussion in this review in terms of the inclusion and exclusion criteria. The flow diagram of article selection is presented in Figure 1.

The seven recruited studies [11-17] that satisfied the methodological criteria for this review are described and summarized next, and the main features of these studies are presented in Table 1.

**Table 1:** Primary Clinical Characteristics of Included Studies Note. *Participants without stroke (i.e., normal controls) were not included as non-PSD patients when we counted sample sizes. ** Chang et al. periodically assessed and recorded depression scores in all stroke patients instead of diagnosing PSD. Abbreviations: PSD, post-stroke depression; BDNF, brain-derived neurotrophic factor; DSM, Diagnostic and Statistical Manual of Mental Disorders [18-19]; GDS-SF, Geriatric Depression Scale-Short Form [20]; HDRS, Hamilton Depression Rating Scale [21].

Study	Sample Size (PSD/Non-PSD patients)*	Stroke Type	Stage of Stroke	Follow-up Time Post-Stroke	BDNF Extraction	Primary Clinical Measure for Depression Diagnosis
Jiménez et al., 2009 [11]	134 (25/109)	Ischemic	Acute	1 month	Serum	DSM-IV [18]
Yang et al., 2011 [12]	100 (37/63)	Ischemic	Acute	2 weeks	Serum	DSM-IV [18]
Zhou et al., 2011 [13]	93 (35/58)	Ischemic	Acute and chronic	6 months	Serum	DSM-IV [18]
Li et al., 2014 [14]	216 (59/157)	Ischemic	Acute and chronic	3 months	Serum	DSM-III-R [19]
Chang et al., 2018 [15]	26 (-/-)**	Ischemic or hemorrhage	Subacute	2 weeks	Serum	GDS-SF [20]
Han et al., 2020 [16]	162 (61/101)	Ischemic or hemorrhage	Acute and chronic	3 weeks	Serum	DSM-IV [18]
Syafrita et al., 2020 [17]	72 (36/36)	Ischemic	Acute	1 month	Serum	HDRS [21]

All seven included research articles were reports of longitudinal observational studies. Jiménez et al. [11] studied 134 patients with a first episode of ischemic stroke and found that serum BDNF levels on around day seven post-stroke were not significantly different in patients with and without PSD at discharge (13.6 ng/ml (9.8-20.1) and 12.9 ng/ml (10.6-16.1), respectively; p = .565); similarly, by day 30, serum BDNF was lower in patients with PSD than in those without, but again, this finding was non-significant (11.4 ng/ml (9.6-19.8) and 13.5 ng/ml (11.1-18.5), respectively; p = .365). Yang et al. [12] studied 100 patients with acute ischemic stroke and 50 healthy control participants and showed that the serum BDNF levels of PSD patients on day one were significantly lower than those in non-PSD patients (p < .001), whereas no significant differences were observed on day seven among the three groups (i.e., PSD patients, non-PSD patients, and healthy controls; p = .064). Furthermore, when the occurrence of PSD was regarded as a dependent variable resulting from different BDNF levels, lower BDNF levels on day one post-stroke were found to be significantly related to increased PSD risk (p = .001) [12]. Zhou et al. [13] studied 112 patients with acute ischemic stroke and 30 healthy controls and showed that the serum BDNF levels in the acute stages of stroke were not significantly different between patients with and without PSD. BDNF changes in non-PSD patients between seven days and six months post-stroke were also non-significant [13]. However, at three to six months post-stroke, serum BDNF was decreased for patients with a confirmed diagnosis of PSD as compared to normal controls and non-PSD patients (p = .035, p = .027, respectively). Li et al. [14] studied 295 patients with ischemic stroke, finding that serum BDNF in PSD patients was significantly decreased at the time of admission compared to non-PSD patients (p < .001). This finding remained significant even when cases of minor depression were included (p < .001). The serum BDNF levels were found to be negatively correlated with scores on the NIH Stroke Scale (NIHSS) [22] (r = - .286, p < .001), so lower serum BDNF levels were associated with increasing severity of stroke. There was no correlation between BDNF level and gender (p = .211) or age (p = .326) [14]. Chang et al. [15] studied 45 patients with subacute stroke and found that serum BDNF levels were significantly and positively correlated with scores on the Geriatric Depression Scale short form (GDS-SF) at each of three time points: before the start of the study, one week, and two weeks after the clinical trial (r = .480, p = .013; r = .477, p = .014; r = .480, p = .013, respectively). No significant association between serum levels of mature BDNF and stroke severity was found [15]. Han et al. [16] studied 162 consecutive stroke patients without any previous history of depression and anxiety and found that a low serum concentration of BDNF was a valuable predictor for the occurrence of PSD. However, using the binary logistic regression model, the odds ratio was only 0.93 (indicating a negative relationship between the categorical and continuous variables), though p = .009. Further, they determined that the risk of PSD development was much higher in ischemic stroke patients who were divorced or separated and who had lower levels of serum BDNF (p = .002) [16]. Syafrita et al. [17] studied 72 post-ischemic stroke patients and found that serum BDNF levels in the acute phase of stroke were significantly lower in PSD patients as compared to non-PSD patients (p = .009), with serum BDNF levels significantly negatively correlated with the risk of PSD development (r = - .308) [17].

A summary of the main findings of reviewed studies is presented in Table 2.

**Table 2:** Main Findings of Included Studies Abbreviations: NA, not available; BDNF, brain-derived neurotrophic factor; PSD, post-stroke depression; GDS-SF, Geriatric Depression Scale – Short Form [20]. *Effect size computed by using Cohen's d [23].

Study	Effect Size* and Significance of Main Results	Main Findings
Jiménez et al., 2009 [11]	NA, Non-significant (p > .05)	No association between BDNF and PSD development was found in patients with a first episode of ischemic stroke at either discharge or one-month follow-up.
Yang et al., 2011 [12]	Large (d = 1.2), Significant (p < .05)	1) Serum BDNF levels of PSD patients on day one were significantly lower than in non-PSD patients; 2) lower BDNF levels on day one were associated with increased PSD risks when PSD was regarded as a dependent variable; 3) by day seven, the difference in BDNF levels between PSD and non-PSD patients was no longer statistically significant.
Zhou et al., 2011 [13]	Small (d = 0.1), Significant (p < .05)	1) In acute stages, there was no significant difference in serum BDNF between non-PSD patients and those who were later diagnosed with PSD; 2) serum BDNF levels of PSD patients were decreased at the time of confirmed diagnosis of PSD (three to six months), compared to normal controls and non-PSD patients.
Li et al., 2014 [14]	Large (d = 1.5), Significant (p < .05)	1) Serum BDNF levels of PSD patients were significantly decreased at the time of admission compared to non-PSD patients; 2) serum BDNF levels were significantly negatively correlated with stroke severity.
Chang et al., 2018 [15]	NA, Significant (p < .05)	1) Serum BDNF levels were significantly positively correlated with GDS-SF score in subacute stroke patients at the start, mid-point, and end of a two-week rehabilitation program; 2) there was no significant association between serum BDNF levels and stroke severity.
Han et al., 2020 [16]	Medium (d = 0.5), Significant (p < .05)	1) Low BDNF concentration was a significant predictor for the occurrence of PSD; 2) social factors (such as being divorced or separated) were also important.
Syafrita et al., 2020 [17]	Medium to large (d = 0.6), Significant (p < .05)	1) BDNF levels were significantly decreased in acute stroke patients with depression compared to those without depression; 2) serum BDNF levels measured during the acute phase of stroke were negatively correlated with risks of PSD development at one month post-stroke.

Finally, five of the seven papers reviewed reported the percentages of PSD and non-PSD patient samples who experienced left vs. right hemisphere strokes (the exceptions were Li et al., 2014 [14], who did not report information about lesion location, and Chang et al., 2018 [15], who did not separate the sample into PSD vs. non-PSD patients). Collapsing the samples together, PSD patients presented with left hemisphere strokes slightly less often than those who did not experience PSD (overall percentages: left hemisphere stroke in non-PSD patients = 43.2%, SD = 10.61 [range 25.4% - 52.8%]; in PSD patients = 41.72%, SD = 10.96 [range = 30.6 - 54.3]). In none of the reviewed studies was lesion location (left vs. right hemisphere) statistically significant in terms of predicting PSD. Only Jimenez et al. (2009) [11] broke lesion location down by gross structure, revealing that most PSD patients had basal ganglia involvement (32.0%) while most non-PSD patients had frontal lobe lesions (32.1%). Other lesion locations reported for both groups were the occipital lobe, posterior fossa, occipital and temporal lobes; only non-PSD patients reported parietal lesions, but there was no lesion location that appeared unique to the PSD group.

Infarct volume was reported in three of the seven papers reviewed [11-12, 14]. Only Yang et al. [12] reported a significant effect of infarct volume associated with PSD, finding that PSD patients had significantly larger lesions than non-PSD patients (PSD mean infarct volume: 3.26ml + 1.61-11.05; non-PSD mean infarct volume: 1.4ml + 0.5-5.54; p = .006). Jimenez et al. [11] and Li et al. [14] reported on patients with much larger lesions than those quantified by Yang et al. [12], but found no significant differences between the PSD and non-PSD groups on this variable (Jimenez et al., PSD mean infarct volume: 9.5ml + 19.9; non-PSD mean infarct volume 9.6ml +13.6; p = .947; Li et al., PSD mean infarct volume = 12.2ml + 1.5; non-PSD mean infarct volume 12.5ml + 1.6; p = .424).

Discussion

Five studies out of the seven included in this review demonstrated a significant correlation between increased risk of PSD development and low BDNF levels at some stage of stroke recovery [12-14,16-17].

However, there are important inconsistencies in the reported data. Jimenez et al. [11] found no statistically significant difference in serum BDNF levels between PSD and non-PSD study participants either early or late in stroke recovery. Yang et al. [12], Li et al. [14], and Syafrita et al. [17] reported lower BDNF in PSD patients than non-PSD patients at acute stages of stroke while Zhou et al. [13] reported no significant differences between BDNF levels in acute stroke patients with or without later-identified PSD. Zhou et al. [13] did report significantly lower BDNF levels in PSD patients at a later stage in the evolution of their stroke (three to six months), but this is contradicted by Yang et al.’s finding that the early significant difference was no longer apparent by day seven post-stroke [12].

Along with these inconsistencies, there are also differences in the reported predictive value and correlations between lower BDNF and risk of PSD. Yang et al. [12] reported that lower BDNF significantly predicted PSD on day one post-stroke but not later in recovery. Li et al. [14] found a significant negative correlation between serum BDNF and scores on the NIHSS during acute phases while Chang et al. [15] showed an opposite effect between serum BDNF and scores on the GDS-SF, significant at all time points (day zero, week one, week two) of their longitudinal study. Both Han et al. [16] and Syafrita et al. [17] reported that reduced BDNF significantly predicts risk of PSD, but Zhen et al. related this to social factors such as divorce while Syafrita et al. commented only on acute phases of stroke recovery.

The inconsistencies extend to observations of lesion location and infarct volume. Only one of the reviewed studies [12] showed a significant difference in infarct volume between PSD and non-PSD patients, with the latter having significantly smaller infarcts. However, for the two other studies reporting infarct volume [11,14], the lesions overall were much larger and the effect was non-significant. Lesion location was inconsistently reported (only in 5 of the 7 papers, and only hemispheric information was reported in 4 of those) and no consistent patterns of association between PSD and lesion location were observable as a result. Hence, it is not possible to determine from the papers reviewed here whether lesion location or infarct volume might play a predictive role in the development of PSD, and more detailed studies with larger samples will be needed to address this question definitively.

Although a high depression scale score does not necessarily equate to the confirmed diagnosis of PSD, at least some of the patients in Chang et al.’s study who scored high on the depression scale could be considered PSD patients, thus making their findings seemingly contradict ones in previous studies. Several explanations for this apparent contradiction are possible. First, Chang et al. performed the assessments of depression scores at the same time as the measurements of serum BDNF levels, in contrast with the five reviewed studies with similar findings (i.e., [12-14,16-17]), wherein serum BDNF was measured early in stroke evolution but depression was evaluated later. Second, the association between serum BDNF levels and various functional impairments (e.g., cognition dysfunction and motor dysfunction) in stroke patients was not taken into consideration by Chang et al. [15]. The lack of assessment for depression at the chronic stroke phase and the absence of statistical adjustment of results based on patient characteristics (e.g., age, sex, and stroke severity) may affect the results and account for apparent differences in findings.

In addition, most of the studies reviewed here did not conduct rehabilitation programs for stroke patients during clinical experiment phases; Chang et al., by contrast, followed a cohort of stroke patients during a standard two-week rehabilitation program. Different types and levels of rehabilitation training could variously affect the expression of BDNF levels in stroke patients. For example, whether patients with low BDNF levels at the early phase of stroke received rehabilitation training, such as physical therapy or psychological support, could lead to different results (PSD vs. non-PSD). Thus, in this case, BDNF concentrations in the early phase of stroke may not accurately predict the risks of PSD development. Methods for evaluating depression also varied between these papers. Marc et al. [24] point out that the GDS-SF, which was used by Chang et al. [15], might tend to underestimate depression since it systematically excludes somatic symptoms [24]; these are included on DSM-IV lists, for instance, which were applied to identify depression in five of the six the other studies reviewed here [11-14,16]. Accordingly, varying methods for diagnosing depression may also account for different results to some degree. More group studies of large patient cohorts, applying similar experimental designs, reporting on rehabilitation procedures, and consistently applying similar assessment protocols, are needed to further clarify the apparent contradictions between these findings.

Apart from clinical trials, associations between BDNF and PSD have also been studied by many researchers from the perspective of genetics. For instance, Kim et al. [25] pointed out that a high BDNF methylation status was correlated with decreased neuronal expression of BDNF and showed that this was independently and significantly associated with PSD incidence. Therefore, although they did not directly explore the relationship between BDNF levels and PSD in patients with stroke, it was indicated that decreased expression of BDNF levels might be associated with the development of PSD [25]. In an earlier study, Kim et al. [26] had reported that the number of Met alleles of BDNF Val66Met polymorphisms correlated with increased PSD incidence. However, Zhou et al. [13] indicated that Val66Met polymorphisms did not correlate with serum BDNF levels. BDNF genotypes (i.e., Val66Met polymorphisms) can vary in different patients, and although this may not be directly related to serum BDNF levels we conjecture that the number of expressed Met alleles likely determines whether or not PSD can be eventually triggered [26]. In other words, the levels of serum BDNF may be similar or even the same in patients with different BDNF genotypes, but patients may develop PSD - or not - depending on their genotype. This suggests that serum BDNF levels at a specific phase of stroke may not be a particularly reliable predictor of later PSD development, and also helps to clarify why the reviewed studies yield apparently contradictory results.

Several lines of evidence exploring the role of BDNF have indirectly indicated that the alteration of serum BDNF levels in stroke patients might play a critical role in the pathogenesis of PSD. BDNF is known to significantly enhance neuroprotective effects in brain pathology [27]. In a preclinical study, Schäbtz et al. [28] showed that BDNF could induce antiapoptotic mechanisms after stroke insults and impede secondary neuronal cell death. Conditional BDNF knockout mice display increased depression-related behaviors, indicating that low expression of BDNF might precipitate depressive disorder [29]. Likewise, Chen et al. [30] showed that low BDNF concentrations in rats resulted in a decrease of neurogenesis in the hippocampus, thereby possibly inducing PSD [30]. BDNF has also been demonstrated to possess antidepressant effects in animal models of depression [31]. These pieces of empirical evidence seem to support the perspective that decreased BDNF concentrations play a crucial role in the pathophysiology of depression. However, as the mechanism of action for BDNF in the development of PSD in human beings remains unclear at present, we still cannot reach a precise conclusion about the association between serum BDNF levels and the development of PSD in human patients.

Caution is warranted when drawing conclusions from any review due to unavoidable limitations. The major limitations of this review were heterogeneity phenomena associated with different studies, and these issues were reflected in variations with respect to experimental design (e.g., degree and content of rehabilitation training post-stroke, time point of depression measurement, duration of clinical follow-up), participants sampled (e.g., whether patients were randomly selected, stage of stroke after admission), and approaches to statistical analysis (e.g., whether potential confounding factors linked to the induction of depression, such as social support and economic status, were considered). In addition, the number of included studies was small, so the findings in this review may not be generalizable to all stroke patients. In fact, as discussed above, contradictory findings regarding the association between changes in BDNF concentration and PSD were reported [11,15], although consistent results were identified from the other five out of seven reviewed papers (i.e., [12-14,16-17]). Thus, further research into the association and predictive role of serum BDNF levels on developing PSD is urgently needed.

Despite these limitations, this review contributes to the discussion of the applicability of BDNF as a biomarker for the early screening of potential PSD patients. The precise nature of the association between changes in BDNF levels and the pathogenesis of PSD remains to be determined, so further studies are needed to explore the role of circulating BDNF in the development of PSD.

This review suggests some concrete issues that can be addressed when future research in this area is conducted. We recommend that the period of observation post-stroke should be lengthened to more accurately determine which patients do and do not manifest PSD during the period of recovery. Also, the timing of serum BDNF level sampling and of post-stroke depression assessments should be considered carefully so that observations can be related to the acute vs. chronic stages of post-stroke recovery and symptom evolution. Additionally, future study results should take account of patient characteristics (especially the lesion size and location), for instance, through the application of statistical weightings to account for stroke severity.

## Conclusions

In conclusion, this review discussed the relationship between BDNF and PSD in detail, suggesting that the changes in serum BDNF concentration can be associated with depression after stroke insults. Inconsistencies in findings and approaches mean that the debate is still open whether serum BDNF levels, especially in the early phase of stroke, can be a potentially effective biomarker for predicting the risk of subsequent PSD development. Hence, in the future, more clinical trials evaluating the relationships between BDNF and PSD should be conducted to draw more reliable conclusions.
